# Lipoprotein subfraction profiling in the search of new risk markers for myocardial infarction: The HUNT study

**DOI:** 10.1371/journal.pone.0285355

**Published:** 2023-05-05

**Authors:** Sigri Bakken Sperstad, Julie Caroline Sæther, Marie Klevjer, Guro Fanneløb Giskeødegård, Tone Frost Bathen, Ragnhild Røsbjørgen, Håvard Dalen, Anja Bye

**Affiliations:** 1 Department of Circulation and Medical Imaging, NTNU, Trondheim, Norway; 2 Department of Biotechnology and Nanomedicine, SINTEF Industry, Trondheim, Norway; 3 Clinic of Cardiology, St. Olavs University Hospital, Trondheim, Norway; 4 Department of Public Health and Nursing, K.G. Jebsen Center for Genetic Epidemiology, Trondheim, Norway; 5 Department of Medicine, Levanger Hospital, Nord-Trøndelag Hospital Trust, Levanger, Norway; Boston University, UNITED STATES

## Abstract

**Background:**

Traditional biomarkers used to measure risk of myocardial infarction (MI) only explain a modest proportion of the incidence. Lipoprotein subfractions have the potential to improve risk prediction of MI.

**Aim:**

We aimed to identify lipoprotein subfractions that were associated with imminent MI risk.

**Methods:**

We identified apparently healthy participants with a predicted low 10-year risk of MI from The Trøndelag Health Survey 3 (HUNT3) that developed MI within 5 years after inclusion (cases, n = 50) and 100 matched controls. Lipoprotein subfractions were analyzed in serum by nuclear magnetic resonance spectroscopy at time of inclusion in HUNT3. Lipoprotein subfractions were compared between cases and controls in the full population (N = 150), and in subgroups of males (n = 90) and females (n = 60). In addition, a sub analysis was performed in participants that experienced MI within two years and their matched controls (n = 56).

**Results:**

None of the lipoprotein subfractions were significantly associated with future MI when adjusting for multiple testing (p<0.002). At nominal significance level (p<0.05), the concentration of apolipoprotein A1 in the smallest high-density lipoprotein (HDL) subfractions was higher in cases compared to controls. Further, in sub analyses based on sex, male cases had lower lipid concentration within the large HDL subfractions and higher lipid concentration within the small HDL subfractions compared to male controls (p<0.05). No differences were found in lipoprotein subfractions between female cases and controls. In sub analysis of individuals suffering from MI within two years, triglycerides in low-density lipoprotein were higher among cases (p<0.05).

**Conclusion:**

None of the investigated lipoprotein subfractions were associated with future MI after adjustment for multiple testing. However, our findings suggests that HDL subfractions may be of interest in relation to risk prediction for MI, especially in males. This need to be further investigated in future studies.

## Background

Coronary artery disease (CAD) is the leading cause of mortality and morbidity worldwide. The number of people at risk will increase due to increasing obesity, diabetes type 2, inactivity, and ageing, implying that this will continue to be a socioeconomic burden in the years to come [1, 2].

Coronary atherosclerosis, the formation of lipid-rich plaque in the arterial walls, is the most prevalent underlying cause of CAD [1]. Known risk factors, including lipid measurements such as high levels of low-density lipoprotein cholesterol (LDL-C) and low levels of high-density lipoprotein cholesterol (HDL-C), fail to fully predict the risk of CAD, and several patients with estimated low risk suffer from myocardial infarction (MI) each year [3, 4]. Thus, there is a clinical need for new biomarkers that could identify patients with subclinical atherosclerosis with greater precision than today.

Conventional lipid measurements do not differentiate between size, density, or concentrations and compositions of lipoproteins. On the other hand, lipidomics, a recently developed research field using high-throughput profiling, can produce comprehensive analysis of cellular lipids. Lipidomics analyses may therefore provide additional information that is missing in today’s evaluation of cardiovascular disease risk [3, 5]. It is well known that the risk of CAD and the lipoprotein profile is sex specific, and a refined lipid analyses may help identify sex specific markers that are necessary to improve risk prediction of CAD. In the present study, we aim to investigate whether lipoprotein subfractions are associated with a 5-year risk of MI in apparently healthy individuals with predicted low 10-year risk of MI, both in the full population and separate for males and females. Furthermore, we aimed to investigate whether lipoprotein subfractions are associated with 2-year risk of MI.

## Materials and methods

### Study design and ethics

This study was approved by the Regional Committee for Medical and Health Research Ethics of central Norway (138187) and The Trøndelag Health Study (HUNT, 2020/18421). All procedures and data handling were performed according to laws and the Declaration of Helsinki, and all participants gave written informed consent.

### Study participants

The Trøndelag Health Survey 3 (HUNT3) is a large population-based health study where blood samples, clinical measurements, and several questionnaires were collected from 50807 participants between the years 2006–2008 [6]. From this selection, we identified 150 eligible patients based on the following criteria: At time of inclusion in the HUNT3 survey, all participants had to be apparently healthy and between 45–74 years. The participants were considered healthy if they did not have any of the following conditions at inclusion; self-reported CVD (including MI, stroke, angina pectoris, heart failure or other reported heart diseases), chronic kidney disease, diabetes mellitus, body mass index (BMI) > 40 and/or cholesterol-lowering medications. In addition, all participants had to have low risk of acute MI or cerebral stroke within the next 10 years after inclusion in the HUNT3 survey, evaluated by the Norwegian risk model, NORRISK 2, for acute MI and cerebral stroke [7]. From this selection, we identified 50 cases with MI, coded I21 by the International Classification of Diseases 10^th^ revision, experienced within 5 years from inclusion in the HUNT3 survey by using data from the local myocardial infarction registry (Helse Nord-Trøndelag). One hundred controls that matched the cases on age and sex and remained healthy for 10 years after inclusion in the HUNT3 survey, were selected. The controls were considered healthy if they did not have any of the following conditions at The Trøndelag Health Survey 4 (HUNT4); self-reported CVD, chronic kidney disease, diabetes, body mass index (BMI) > 40, and/or use of cholesterol-lowering medication. A flowchart of the inclusion process can be viewed in S1 Fig. From this selection, sub analyses were performed in females (cases = 20, controls = 40), males (cases = 30, controls = 60), and in 2-year MI risk (cases = 19, controls = 38).

### Measurements performed by The HUNT study

At the time of inclusion in the HUNT3 survey, weight and height of the participants were measured on a combined scale (Model DS-102, Arctic Heating AS, Nøtterøy, Norway), BMI was calculated as weight divided by height squared (kg/m^2^), and blood pressure and resting heart rate were measured by established guidelines in a sitting position^14^ (Critikon Dinamap 845XT, GE Medical Systems, Lille Chalfont, Buckinghamshire, United Kingdom).

### Blood sampling by The HUNT study

Biochemical analyses were performed on fresh venous blood samples at Levanger Hospital, Norway. Non fasting glucose (mmol/L) was analyzed by Hexokinase/G-G-PDH methodology (reagent kit 3L82-20/3L82-40 Glucose, Abbot, Clinical Chemistry, USA), HDL-cholesterol (mmol/L) was analyzed by Accelerator selective detergent methodology (reagent kit 3K33-20 Ultra HDL, Abbot, Clinical Chemistry, USA), triglycerides (mmol/L) was analyzed by Glycerol Phosphate Oxidase methodology, and LDL-cholesterol was calculated using the Friedewald formula (21). Remaining serum samples were stored at -80°C in the HUNT biobank. In the present study, 250 μL frozen serum from each participant were used for lipoprotein subfraction analysis.

### Nuclear magnetic resonance spectroscopy

Serum samples were analyzed for lipoproteins and lipoprotein subfractions by nuclear magnetic resonance (NMR) spectroscopy at the MR Core Facility at the Norwegian University of Science and Technology in Trondheim, Norway. 120 μL serum was mixed with 120 μL buffer (20% D_2_O with 0.075 M Na_2_HPO_4_, 6 mM NaN_3_, 4,6 mM trimethylsilylpropanoic acid, pH 7.4) and transferred to 3-mm NMR tubes. The analyses were performed on Bruker Avance III UltraShield Plus 600 MHz spectrometer (Bruker BioSpin) equipped with a 5mm broad band inverse (BBI) probe. Further procedures were fully automated using a SampleJet with Icon-NMR on Topspin 3.6.2 (Bruker BioSpin). 1D 1H Nuclear Overhauser effect spectroscopy (NOESY) and Carr–Purcell–Meiboom–Gill (CPMG) spectra with water presaturation were obtained at 310 K using acquisition and processing parameters similar to Dona et al. [8]. The automated Bruker IVDr Lipoprotein Subclass Analysis (B.I.LISA^TM^) method was used to quantify the lipoprotein parameters (23). This method estimated the concentrations of cholesterol, free cholesterol, phospholipids, apolipoprotein A1 (Apo-A1), apolipoprotein A2 (Apo-A2) and apolipoprotein B (Apo-B) in total serum, as well as in each of the lipoproteins (very-low-density lipoprotein (VLDL), intermediate-density lipoprotein (IDL), low-density lipoprotein (LDL), and high-density lipoprotein (HDL)). Each lipoprotein was further divided into subfractions according to their density; VLDL into VLDL-1 to VLDL-5, LDL into LDL-1 to LDL-6, and HDL into HDL-1 to HDL-4, with increasing density (S1 Table). The concentrations of triglycerides, cholesterol, free cholesterol, phospholipids, Apo-A1, Apo-A2 and Apo-B were extracted for each lipoprotein subfraction. Particle numbers for total serum, VLDL, IDL, LDL and in LDL-1-6 were also extracted. In total, the NMR spectroscopy analysis yielded a dataset of 112 variables for each sample. S2 Table and S2 Fig show an overview of all measured variables.

### Statistical analyses

Statical analyses were performed by using SPSS Statistics version 27.0 (IBM SPSS, New York, USA) and MATLAB R2017a with PLS_Toolbox 8.2.1 (Eigenvector Research, Inc.). Principal Component Analyses (PCA) were performed on autoscaled lipoprotein concentrations to find possible outliers in the dataset. The Shapiro Wilk test of normality were conducted to check for normally distributed data. None of the lipoprotein subfractions nor other clinical variables were normally distributed, and the Mann Whitney U test were therefore used to compare both clinical characteristics and lipoprotein subfractions between cases and controls. Partial Least Squares Discriminant Analysis (PLS-DA) were applied to compare differences in lipoprotein profile between cases and controls in the full population and separately for males and females. PLS-DA were performed on autoscaled variables and validated by 10-fold cross validation with 20 iterations. Premutation testing with 1000 permutations was performed to determine the significance of the final model. The threshold for nominal significant differences between cases and controls were set to p-value < 0.05. The false discovery rate for the Benjamini-Hochberg test was set to 0.05, resulting in a multiple tested corrected p < 0.002. Continuous data are presented as mean ± standard deviation (SD) and categorical data as counts with percentages.

## Results

### Characteristics of the study population

Descriptive characteristics of the participants (N = 150) at time of inclusion in the HUNT3 survey are presented in Table 1. None of the registered cardiovascular disease (CVD) risk factors were significantly different between cases and controls. Mean blood pressure, HDL-C and triglycerides were within the recommended range, whereas mean total cholesterol was slightly elevated in both groups. Both groups showed BMI values of 27, equivalent to overweight. Standard laboratory measurements of serum HDL-C and triglycerides from the HUNT3 survey were compared to measurements of HDL-C and triglycerides from NMR spectroscopy to assess internal validity, showing high internal validity (S3 Fig) No outliers were detected in the dataset (S4 Fig).

**Table 1:** Characteristics of the participants (N = 150).

Characteristics	Cases (n = 50)	Controls (n = 100)
Sex (female/male)	20/30	40/60
Age (years)	56 ± 6	56 ± 5
Body mass index (kg/m^2^)	27.1 ± 3.8	27.0 ± 3.5
Systolic blood pressure (mmHg)	126 ± 14	126 ± 13
Diastolic blood pressure (mmHg)	75 ± 10	74 ± 9
Total cholesterol (mmol/L)	6.1 ± 0.9	5.9 ± 0.7
High-density lipoprotein cholesterol (mmol/L)	1.3 ± 0.4	1.4 ± 0.3
Triglycerides (mmol/L)	1.6 ± 0.7	1.6 ± 0.8
Non-fasting glucose (mmol/L)	5.8 ± 1.1	5.5 ± 0.9
Time since last meal (hours)	3 ± 2	2 ± 2
Medically treated hypertension	7 (16%)	3 (3%)
Smoker, current or previous	33 (66%)	53 (53%)
First degree relative suffering from myocardial infarction before the age of 60 years	12 (32%)	14 (16%)

Data are presented as mean ± standard deviation or number and percentages.

### Lipoprotein subfractions and 5-year MI risk

**Fig 1:**
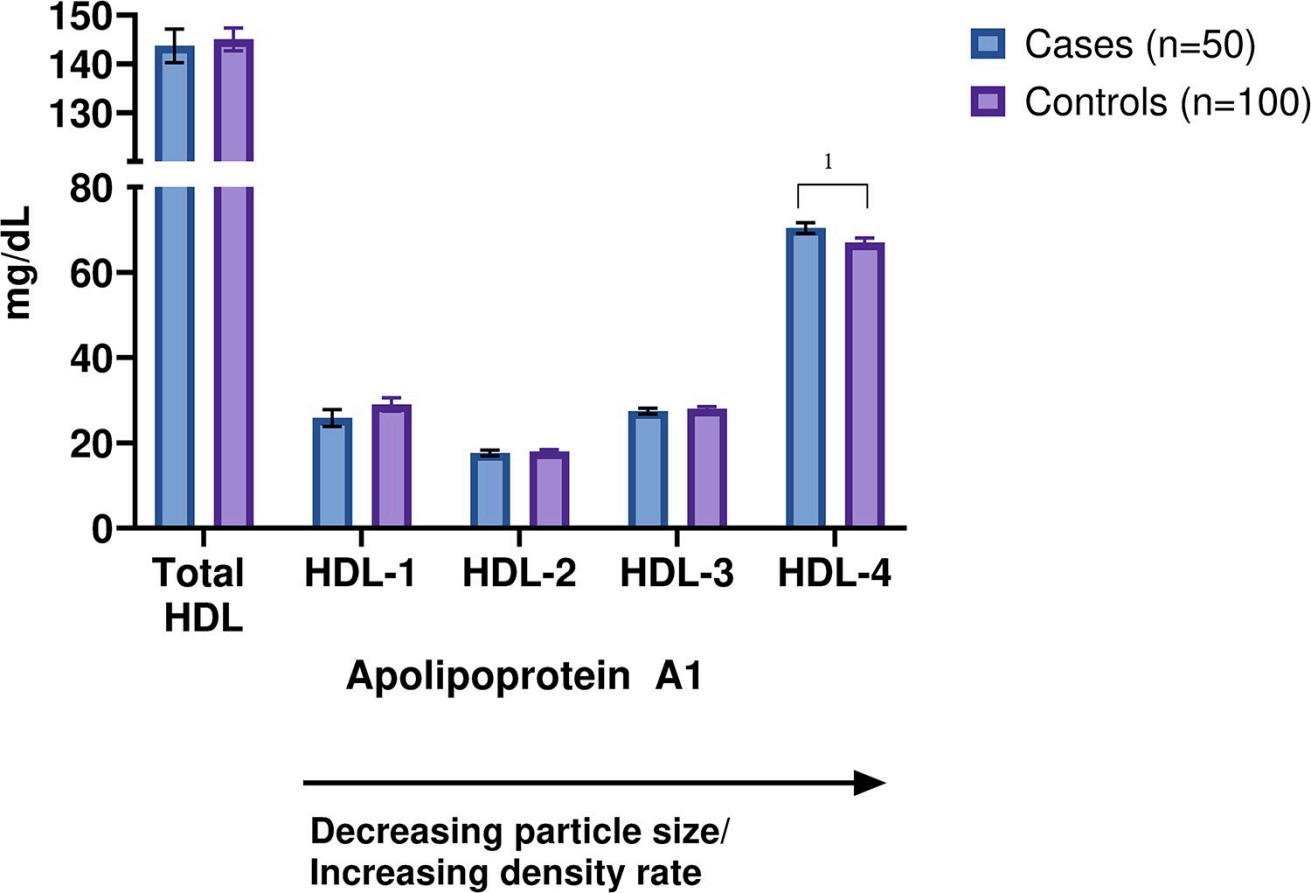
Measurements of Apo-A1 in total HDL and in all HDL subfractions (HDL-1 to HDL-4) among cases (n = 50) and controls (n = 100). Boxplots illustrating median (middle line) and mean (+) with upper quartile, lower quartile, minimum value, and maximum value. *Significant at p < 0.05. Apo-A1, apolipoprotein A1; HDL, high-density lipoprotein.

At time of inclusion in the HUNT3 survey, when all 150 participants were apparently healthy and had a predicted low 10-year risk of MI, none of the lipoprotein subfractions were significantly associated with 5-year MI risk after adjustment for multiple testing. However, at nominal significance level (p < 0.05), the concentration of Apo-A1 in the smallest HDL subfractions (HDL-4) was higher in cases compared to controls (p = 0.03). Fig 1 demonstrates that the elevated concentration of Apo-A1 in HDL-4 in cases is not reflected by measurements of Apo-A1 in the total HDL, potentially masked by the lower levels of Apo-A1 within the larger HDL subfractions. Extended results, including all lipoprotein subfractions, are listed in S3 Table.

### Subgroup analyses based on sex

**Fig 2:**
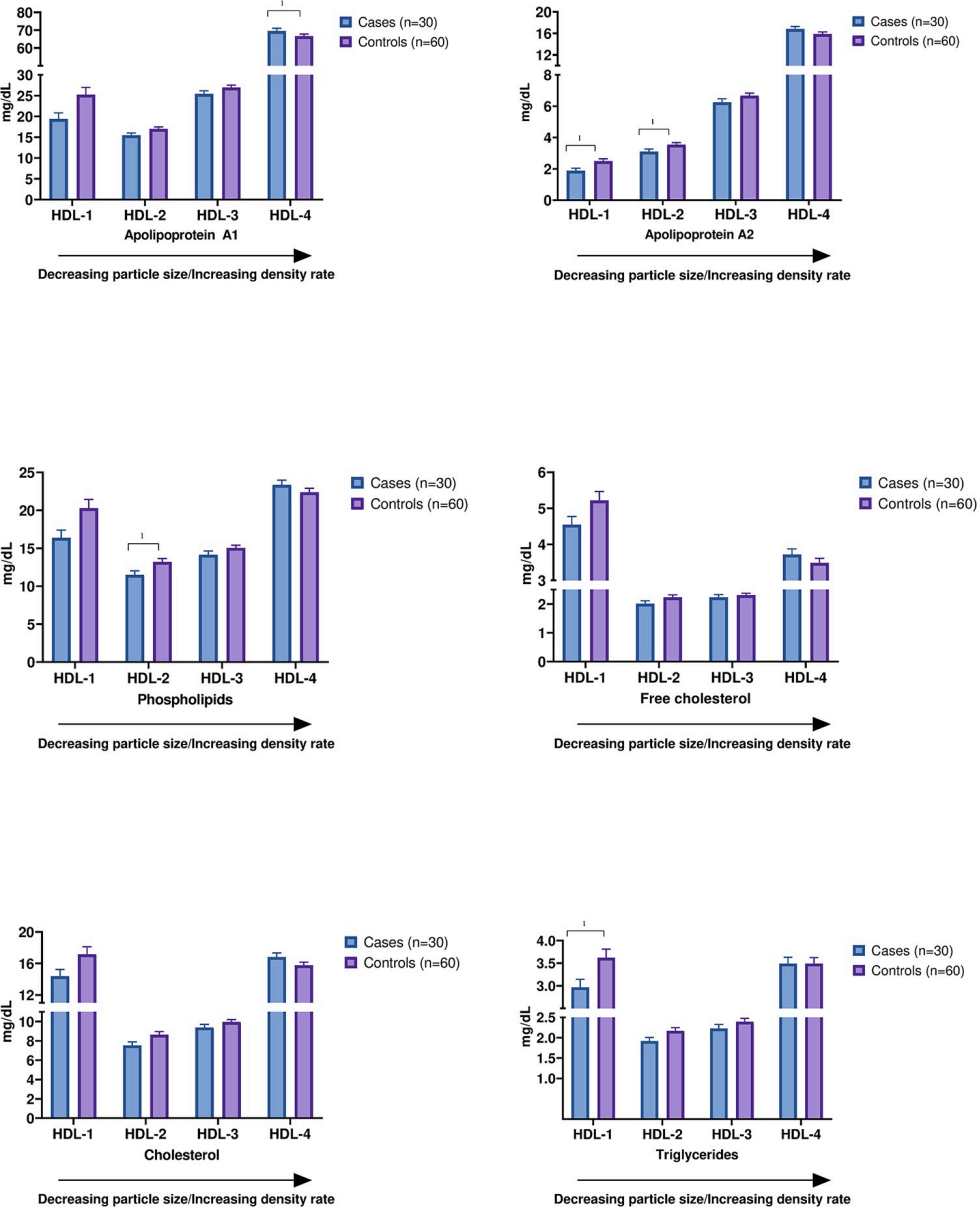
Measurement of Apo-A1, Apo-A2, phospholipids, free cholesterol, cholesterol, and triglycerides in all HDL subfractions (HDL-1 to HDL-4) among male cases (n = 30) and male controls (n = 60). Boxplots illustrating median (middle line) and mean (+) with upper quartile, lower quartile, minimum value, and maximum value. *Significant at p < 0.05. Apo-A1, apolipoprotein A1; Apo-A2, apolipoprotein A2; HDL, high-density lipoprotein.

From the PLS-DA, the lipid profile of male and females were significantly different (p < 0.001, S5 Fig), and we therefore performed sub analyses based on sex. Our results showed no significant differences in lipoprotein subfractions between female cases and controls (n = 60), but five HDL subfractions were nominally significantly different between male cases and controls (n = 90, p < 0.05, Fig 2). Similar to the results in the full population, the Apo-A1 in HDL-4 was found to be elevated in cases compared to controls. In large HDL-subfractions, the concentrations of triglycerides and Apo-A2 in HDL-1 and phospholipids and Apo-A2 in HDL-2 were reduced in cases compared to controls (Fig 2). No difference was found between male cases and controls in concentration of cholesterol and free cholesterol in HDL subfractions. There seems to be a trend that the HDL subfractions 1–3 are reduced in cases, whereas the small HDL-4 subfractions are elevated in cases compared to controls (Fig 2). Small and large HDL particles may therefore possess opposite association with future MI risk. Extended results for males, including all lipoprotein subfractions, are listed in S4 Table.

### Subgroup analysis, lipoprotein subfractions and 2-year MI risk

The cases experienced MI within 5 years despite a predicted low risk of MI at the time of inclusion in the HUNT3 survey. Participants that experienced MI within 2 years are of particular interest as these are assumed to have a more pronounced risk profile. Therefore, a subgroup analysis was performed on participants that experienced MI within 2 years (N = 19) and their matched controls (n = 38). None of the lipoprotein subfractions were significantly associated with 2-year risk of MI when adjusting for multiple testing. However, cases had 20% higher concentration of triglycerides in total LDL (LDTG) compared to controls at nominal significance level (p < 0.05). Extended results, including all lipoprotein subfractions, are listed in S5 Table.

## Discussion

In the present study, we addressed a clinically important group of individuals that were mistakenly classified as low-risk individuals when they should have been classified as high-risk individuals by the risk prediction model NORRISK 2. Biomarkers that can identify these individuals may add value to the current risk prediction models, which would be of great clinical and socioeconomic importance. Thus, we quantified a large number of lipoprotein subfractions in serum samples from 150 apparently healthy individuals with a predicted low 10-year risk of MI at the time of inclusion in the HUNT3 survey. The selected cases (n = 50) experienced MI within 5 years after the blood sampling at the time of inclusion in The HUNT3 Survey, and the matched controls (n = 100) remained healthy during the 10-year observation period. We investigated the association between lipoprotein subfractions and a 5-year risk of MI. None of the analyzed lipoprotein subfractions were significantly associated with future MI after adjusting for multiple testing, but some passed the nominal significance threshold.

Quantification of circulating Apo-A1 is occasionally performed in the clinical setting, but it is currently not a part of standard CVD risk evaluation [9]. In the present study, there were indications of increased concentrations of Apo-A1 in the smallest HDL particles (HDL-4) in cases compared to controls, despite the presumed cardioprotective role of Apo-A1 and HDL [10]. There were no differences in the concentration of Apo-A1 in total HDL between the groups. This is in line with the assumption that not all HDL subfractions have atheroprotective properties [11, 12]. Our study suggests that Apo-A1 in HDL-4 may have a concealed proatherogenic effect and that elevated Apo-A1 concentrations in the smallest HDL subfractions may not be an advantage for cardiovascular health. Apo-A1 in HDL-4 might be a potential biomarker of MI risk, but future investigations involving larger studies are needed to verify this assumption.

Results from the multivariate analysis, PLS-DA, showed a significant difference in lipidomic profile of males and females. Previous studies have also demonstrated sex-specific differences in lipidomic risk profile and it is generally known that CVD risk differ between sexes [13]. In sex-specific sub analyses, none of the lipoprotein subfractions were significantly associated with MI after adjustment for multiple testing, but some passed the nominal significance threshold (p < 0.05). This must be taken into consideration when interpreting our results. In males, five lipoprotein subfractions were different between cases and controls at nominal significance threshold. This included elevated concentration of Apo-A1 in HDL-4, reduced concentration of triglycerides and Apo-A2 in HDL-1, and reduced concentration of phospholipids and Apo-A2 in HDL-2 among the cases. These findings indicate that elevated lipid concentration within the small HDL particles as well as reduced lipid concentration within the large HDL particles increases the risk of MI. This is in line with previous studies, suggesting that the size of the HDL particles is of importance in CVD risk prediction, and that large particles may have antiatherogenic properties, whereas small particles may have atherogenic properties [14–16].

Previous studies show that large HDL subfractions are negatively associated with CVD risk, possibly due to the assumed atheroprotective effect of large HDL particles and/or due to the low amount of large HDL particles [10, 16, 17]. It is also generally accepted that cholesterol concentration within the different groups of lipoproteins is associated with CVD risk [18]. However, the associations between CVD risk and the concentration of other lipids within lipoprotein subfractions, including phospholipid, free cholesterol, and Apo-A2, are less studied. Previous studies have debated whether Apo-A2 is an anti- or proatherogenic protein, emphasizing that its role in lipoprotein metabolism is still unknown [19]. Phospholipids and free cholesterol are believed to influence the anti-oxidative properties of HDL, but their mechanisms are unidentified [18]. A previous study investigated the concentration of triglyceride and cholesterol within HDL subfractions and its relation to MI risk by using NMR spectroscopy [17]. They found that triglycerides in all HDL subfractions were positively associated with MI risk, and that cholesterol concentration in medium and large HDL particles were inversely associated to MI risk. This is contrary to our findings, as we found a negative association between triglycerides in large HDL-1 and HDL-2 and risk of MI, and no association between cholesterol in HDL subfractions and risk of MI. Although, it has previously been suggested that high levels cholesterol in large HDL particles are decreases risk of CVD and that high levels of cholesterol in the small HDL particles increases risk [11, 20].

In the sub analyses of apparently healthy cases with a predicted low 10-year risk of MI that experienced MI within 2 years, none of the quantified lipoprotein subfractions were significantly different between cases and controls at time of inclusion in the HUNT3 survey. However, at nominally significance threshold (p < 0.05), the concentration of triglycerides in LDL were elevated in cases compared to controls. This is in line with several studies showing a positive association between LDL triglycerides and incident of CVD [21, 22]. It is suggested that this association is independent of LDL cholesterol levels [22]. Triglyceride concentration in blood has also been suggested as a causal risk factor for CVD [23, 24], and LDL triglycerides has previously shown to improve risk prediction of cardiovascular mortality [25]. In the present study, the total concentration of triglycerides in blood were not different between cases and controls, strengthening the relevance of measuring triglycerides of LDL in CVD risk assessment instead of total triglycerides.

To minimize the limitations that comes with a small number of cases available, we included two controls per included case. Still, no significant differences in lipoprotein subfractions between cases and controls were found after adjustment for multiple testing by the false discovery rate approach. This might be explained by the relatively small study population compared with the large number of lipoprotein subfractions measured by NMR spectroscopy. It is suggested that multiple testing is too conservative for lipidomic analyses (39), and a standardized approach are needed to address this issue. Furthermore, to be able to implement lipoprotein subfraction into future clinical risk evaluation, a standardized methods for lipoprotein subfraction analysis is required. None of the participants reported use of cholesterol-lowering medication at the time of blood sampling in the HUNT3 survey through self-reported information in the HUNT4 survey, including questions about current use of cholesterol-lowering medications (yes/no) and first prescription of the medication (age). All controls reported no use of the cholesterol-lowering medication throughout the 10-year observation period, and cases reported to have started medication subsequently after the event of MI. However, this is self-reported information and should have been verified by the Norwegian Prescription Database. Another limitation in our study is that we included six cases with MI coded as I21.9 by the International Classification of Diseases 10^th^ revision. The underlying cause of MI is unspecified in these cases, and we can therefore not be certain that MI is caused by atherothrombosis. This may have influenced our results.

## Conclusion

In the present study, none of the analyzed lipoprotein subfractions were significantly associated with future MI in apparently healthy individuals with a predicted low 10-year risk of MI after adjusting for multiple testing, but some passed the nominal significance threshold. Hence, there were indications that elevated concentration of Apo-A1 in the small HDL-4 subfractions were associated with a 5-year risk of MI, and that elevated concentration of triglycerides in LDL were associated with a 2-year risk of MI. In sub analyses on sex, HDL subfractions were found as potential sex-specific risk markers of MI in males. Compared to male controls, there were indications that male cases had elevated concentration of Apo-A1 in the small HDL-4 subfraction, and reduced levels of triglycerides, Apo-A2, and phospholipids within the large HDL subfractions (HDL-1 and HDL-2). The present study therefore suggests that lipoprotein subfractions may provide valuable information about MI risk. However, the statistical strength of this study is limited by the small study population. Larger studies are therefore needed to validate our findings and to assess the clinical relevance of lipoprotein subfractions in risk prediction of MI.

## Supporting information

S1 Fig Flowchart of the inclusion process. (TIF)

S2 Fig Visual overview of all variables measured by nuclear magnetic resonance spectroscopy. (TIF)

S3 Fig Validation of nuclear magnetic resonance spectroscopy analysis data. (TIF)

S4 Fig Principal component analyses plot. (TIF)

S5 Fig Partial least squares discriminant analysis plots retrieved from nuclear magnetic resonance spectroscopy data. (TIF)

S1 Table Density ranges of lipoproteins and lipoprotein subfractions. (DOCX)

S2 Table Table overview of all variables measured by nuclear magnetic resonance spectroscopy. (DOCX)

S3 Table Comparison of lipid variables between cases and controls (N = 150). (DOCX)

S4 Table Comparison of lipid variables levels between male cases and male controls (n = 90). (DOCX)

S5 Table Comparison of lipid variables between cases and controls in a 2-year observation period (n = 57). (DOCX)
