# Supplementary figures and images for: Lipoprotein subfraction profiling in the search of new risk markers for myocardial infarction: The HUNT study
Source: PLoS One. 2023 May 5;18(5):e0285355. doi: 10.1371/journal.pone.0285355 (PMC10162525; doi:10.1371/journal.pone.0285355)

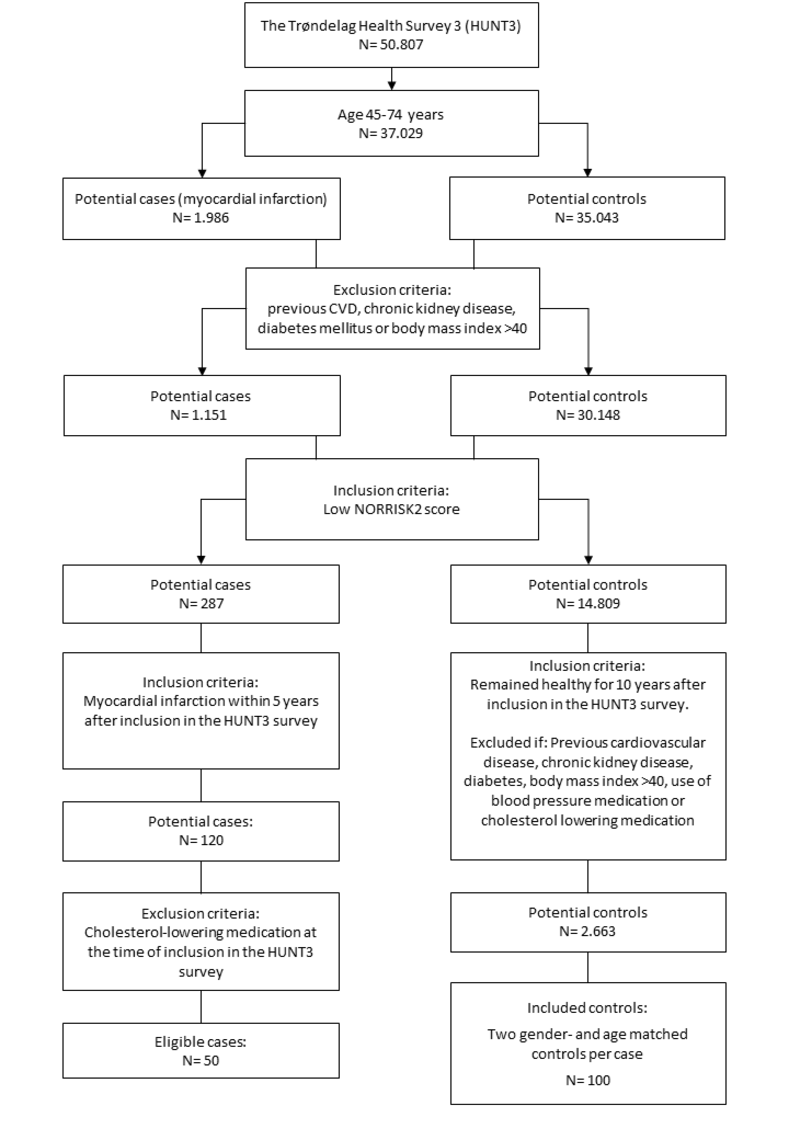

Supplement: S1 Fig — (TIF) [file pone.0285355.s001.tif]

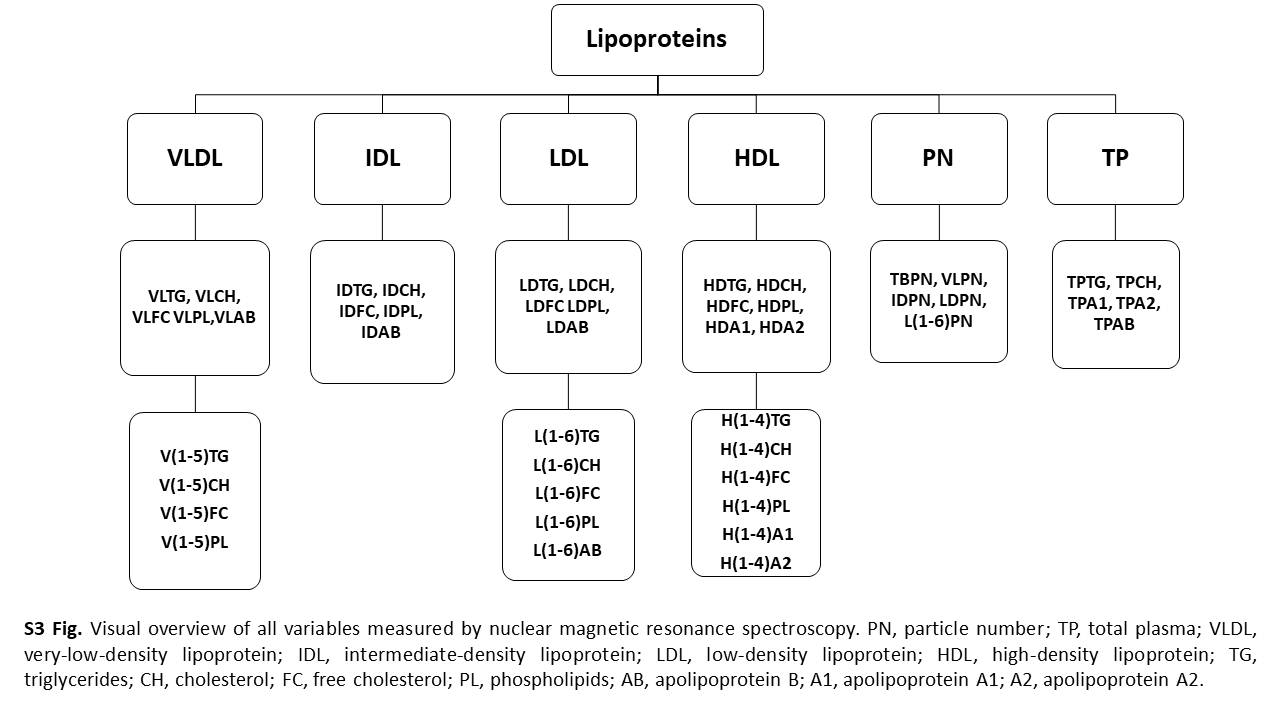

Supplement: S2 Fig — (TIF) [file pone.0285355.s002.tif]

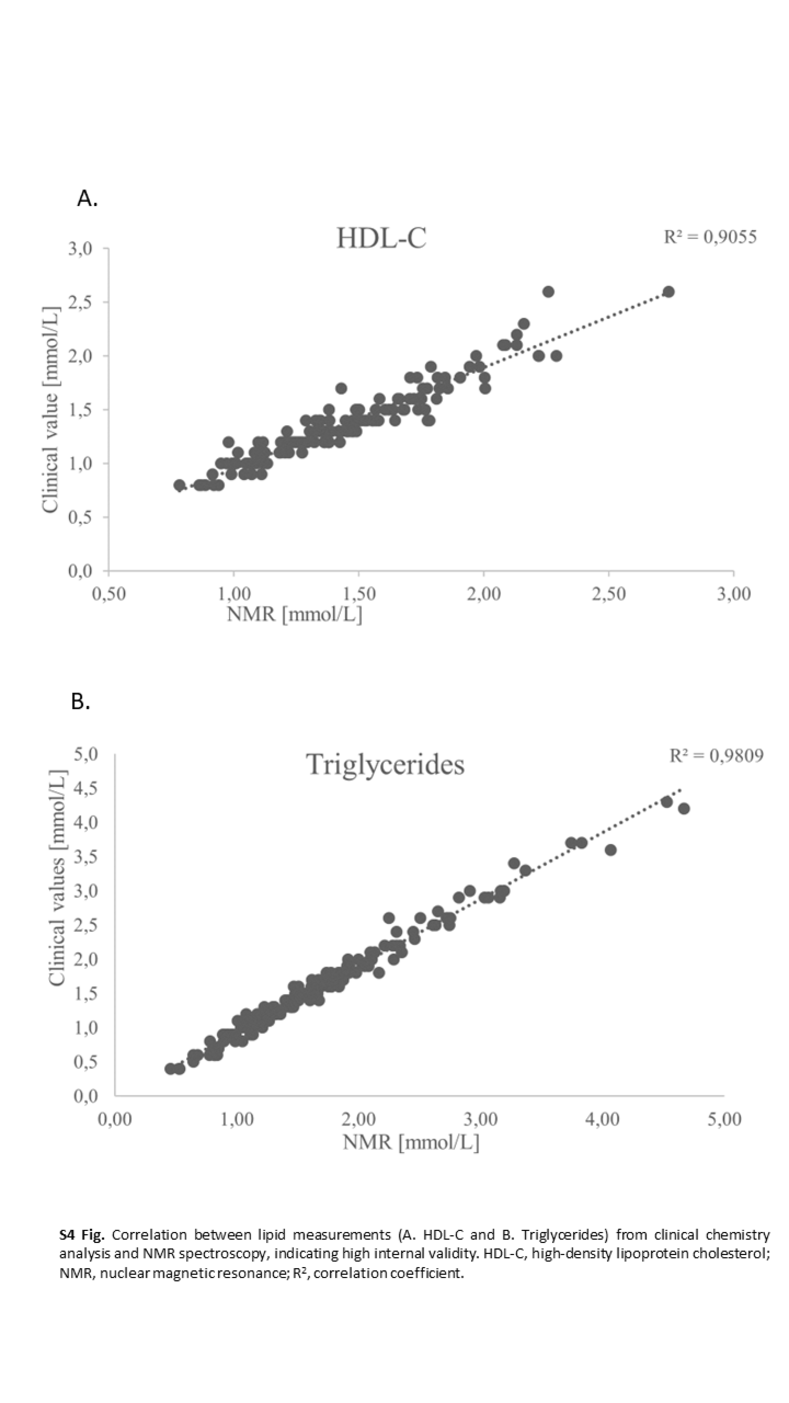

Supplement: S3 Fig — (TIF) [file pone.0285355.s003.tif]

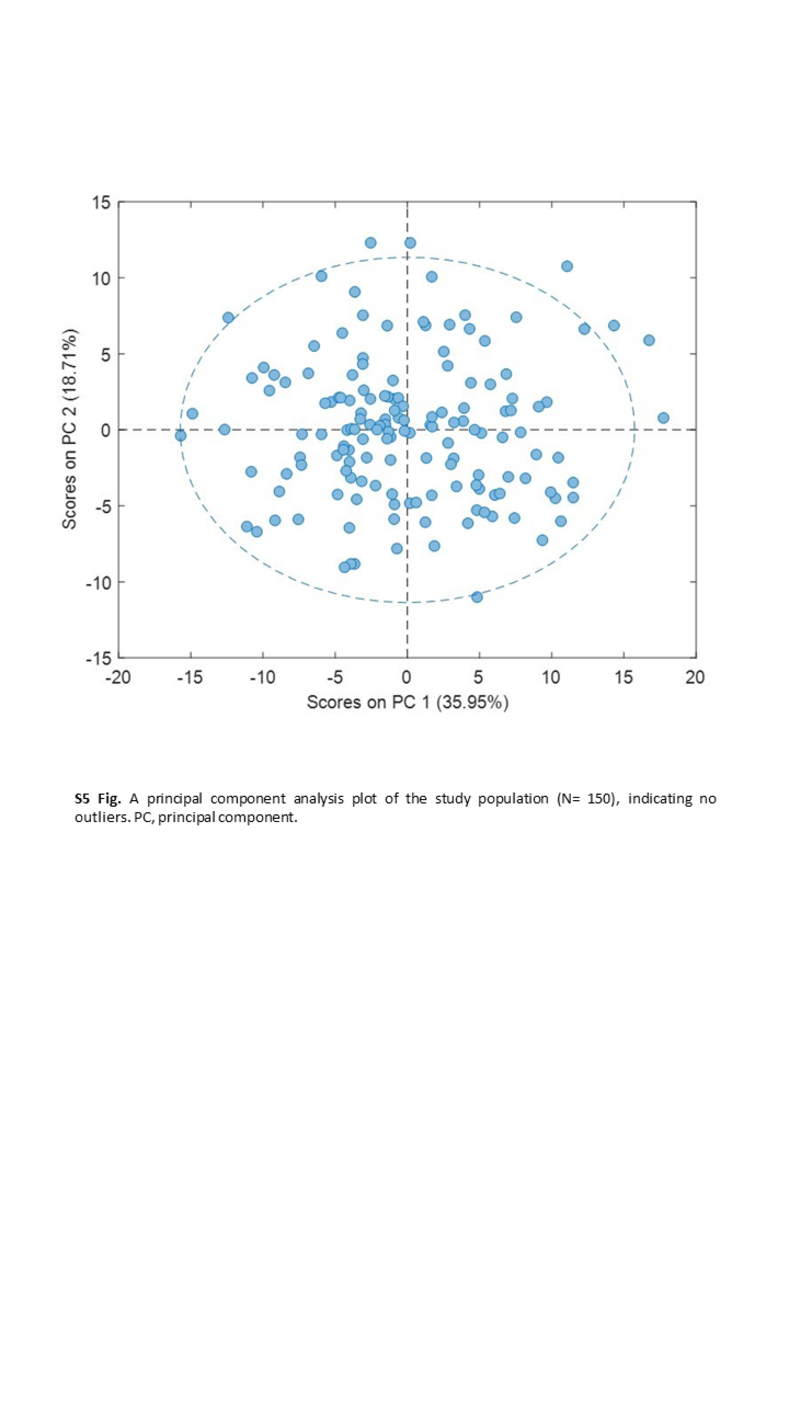

Supplement: S4 Fig — (TIF) [file pone.0285355.s004.tif]

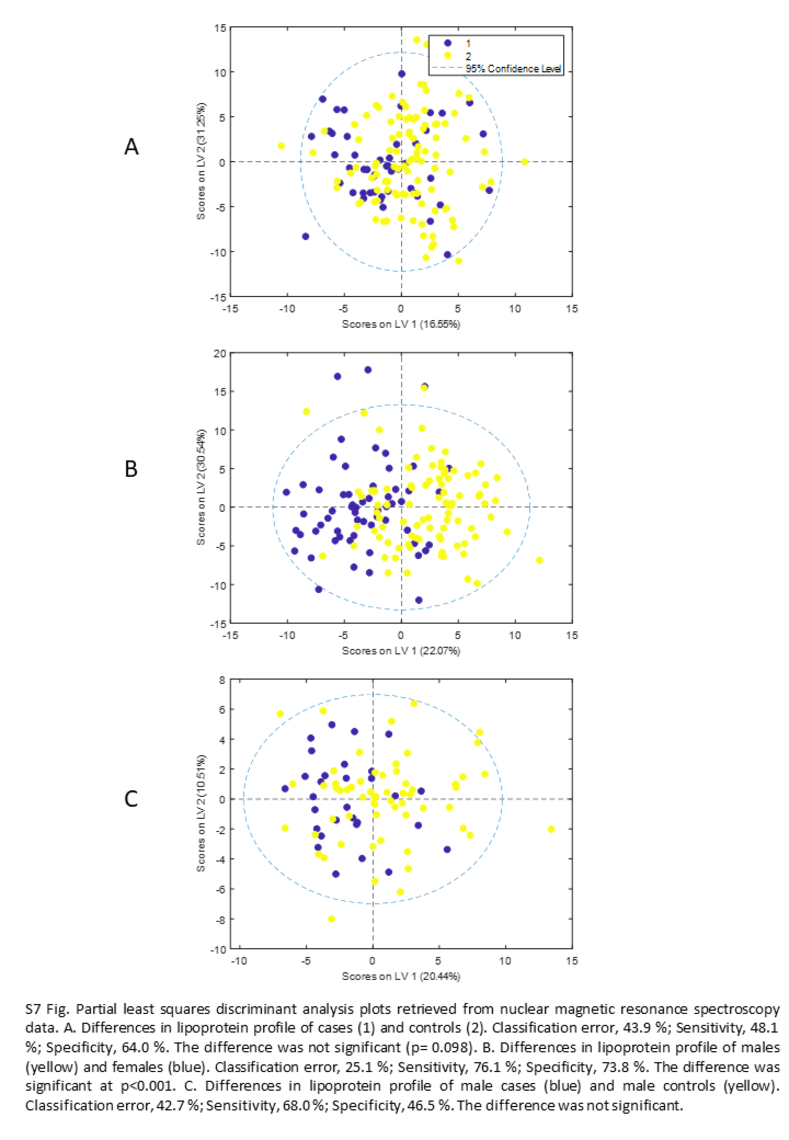

Supplement: S5 Fig — (TIF) [file pone.0285355.s005.tif]
