# Supplementary material for: Lipoprotein subfraction profiling in the search of new risk markers for myocardial infarction: The HUNT study
Source: PLoS One. 2023 May 5;18(5):e0285355. doi: 10.1371/journal.pone.0285355 (PMC10162525; doi:10.1371/journal.pone.0285355)
Supplement: S1 Table — (DOCX) [file pone.0285355.s006.docx]

**S2 Table.** Density ranges of lipoproteins

and lipoprotein subfractions

|  | Range, kg/L |
| --- | --- |
| *Main lipoprotein fractions* | |
| LDL | 1.019-1.063 |
| VLDL | 0.950-1.006 |
| IDL | 1.006-1.019 |
| HDL | 1.063-1.210 |
| *Low-density lipoprotein subfractions* | |
| LDL-1 | 1.019-1.031 |
| LDL-2 | 1.031-1.034 |
| LDL-3 | 1.034-1.037 |
| LDL-4 | 1.037-1.040 |
| LDL-5 | 1.040-1.044 |
| LDL-6 | 1.044-1.063 |
| *High-density lipoprotein subfractions* | |
| HDL-1 | 1.063-1.100 |
| HDL-2 | 1.100-1.112 |
| HDL-3 | 1.112-1.125 |
| HDL-4 | 1.125-1.210 |

LDL, Low-density lipoprotein; VLDL, very-low-density lipoprotein; IDL, intermediate-density lipoprotein; HDL, high-density lipoprotein. The density ranges for very-low-density lipoprotein subfractions 1 to 5 are specified in Lindgren FT, Jensen LL, Hatch FT (1972). The isolation and quantitative analysis of serum lipoproteins. In Nelson GJ (ed.) Blood lipids and lipoproteins: Quantitation, composition and metabolism. Wiley-Interscience, New York, p 181-274.
