# Supplementary material for: Lipoprotein subfraction profiling in the search of new risk markers for myocardial infarction: The HUNT study
Source: PLoS One. 2023 May 5;18(5):e0285355. doi: 10.1371/journal.pone.0285355 (PMC10162525; doi:10.1371/journal.pone.0285355)
Supplement: S2 Table — (DOCX) [file pone.0285355.s007.docx]

**S3 Table.** Table overview of all variables measured by nuclear magnetic resonance spectroscopy

| **Variable** | **Matrix** | **Analyte** | **Unit** |
| --- | --- | --- | --- |
| TPTG | Triglyceride | Total plasma | mg/dL |
| TPCH | Cholesterol | Total plasma | mg/dL |
| LDCH | Cholesterol | LDL | mg/dL |
| HDCH | Cholesterol | HDL | mg/dL |
| TPA1 | Apo-A1 | Total plasma | mg/dL |
| TPA2 | Apo-A2 | Total plasma | mg/dL |
| TPAB | Apo-B | Total plasma | mg/dL |
| LDHD | LDL-C/HDL-C | Total plasma | - |
| ABA1 | Apo-B/Apo-A1 | Total plasma | - |
| TBPN | Total particle number | Total plasma | nmol/L |
| VLPN | Particle number | VLDL | nmol/L |
| IDPN | Particle number | IDL | nmol/L |
| LDPN | Particle number | LDL | nmol/L |
| L1PN | Particle number | LDL-1 | nmol/L |
| L2PN | Particle number | LDL-2 | nmol/L |
| L3PN | Particle number | LDL-3 | nmol/L |
| L4PN | Particle number | LDL-4 | nmol/L |
| L5PN | Particle number | LDL-5 | nmol/L |
| L6PN | Particle number | LDL-6 | nmol/L |
| VLTG | Triglyceride | VLDL | mg/dL |
| IDTG | Triglyceride | IDL | mg/dL |
| LDTG | Triglyceride | LDL | mg/dL |
| HDTG | Triglyceride | HDL | mg/dL |
| VLCH | Cholesterol | VLDL | mg/dL |
| IDCH | Cholesterol | IDL | mg/dL |
| VLFC | Free cholesterol | VLDL | mg/dL |
| IDFC | Free cholesterol | IDL | mg/dL |
| LDFC | Free cholesterol | LDL | mg/dL |
| HDFC | Free cholesterol | HDL | mg/dL |
| VLPL | Phospholipids | VLDL | mg/dL |
| IDPL | Phospholipids | IDL | mg/dL |
| LDPL | Phospholipids | LDL | mg/dL |
| HDPL | Phospholipids | HDL | mg/dL |
| HDA1 | Apo-A1 | HDL | mg/dL |
| HDA2 | Apo-A2 | HDL | mg/dL |
| VLAB | Apo-B | VLDL | mg/dL |
| IDAB | Apo-B | IDL | mg/dL |
| LDAB | Apo-B | LDL | mg/dL |
| V1TG | Triglycerides | VLDL-1 | mg/dL |
| V2TG | Triglycerides | VLDL-2 | mg/dL |
| V3TG | Triglycerides | VLDL-3 | mg/dL |
| V4TG | Triglycerides | VLDL-4 | mg/dL |
| V5TG | Triglycerides | VLDL-5 | mg/dL |
| V1CH | Cholesterol | VLDL-1 | mg/dL |
| V2CH | Cholesterol | VLDL-2 | mg/dL |
| V3CH | Cholesterol | VLDL-3 | mg/dL |
| V4CH | Cholesterol | VLDL-4 | mg/dL |
| V5CH | Cholesterol | VLDL-5 | mg/dL |
| V1FC | Free cholesterol | VLDL-1 | mg/dL |
| V2FC | Free cholesterol | VLDL-2 | mg/dL |
| V3FC | Free cholesterol | VLDL-3 | mg/dL |
| V4FC | Free cholesterol | VLDL-4 | mg/dL |
| V5FC | Free cholesterol | VLDL-5 | mg/dL |
| V1PL | Phospholipids | VLDL-1 | mg/dL |
| V2PL | Phospholipids | VLDL-2 | mg/dL |
| V3PL | Phospholipids | VLDL-3 | mg/dL |
| V4PL | Phospholipids | VLDL-4 | mg/dL |
| V5PL | Phospholipids | VLDL-5 | mg/dL |
| L1TG | Triglycerides | LDL-1 | mg/dL |
| L2TG | Triglycerides | LDL-2 | mg/dL |
| L3TG | Triglycerides | LDL-3 | mg/dL |
| L4TG | Triglycerides | LDL-4 | mg/dL |
| L5TG | Triglycerides | LDL-5 | mg/dL |
| L6TG | Triglycerides | LDL-6 | mg/dL |
| L1CH | Cholesterol | LDL-1 | mg/dL |
| L2CH | Cholesterol | LDL-2 | mg/dL |
| L3CH | Cholesterol | LDL-3 | mg/dL |
| L4CH | Cholesterol | LDL-4 | mg/dL |
| L5CH | Cholesterol | LDL-5 | mg/dL |
| L6CH | Cholesterol | LDL-6 | mg/dL |
| L1FC | Free cholesterol | LDL-1 | mg/dL |
| L2FC | Free cholesterol | LDL-2 | mg/dL |
| L3FC | Free cholesterol | LDL-3 | mg/dL |
| L4FC | Free cholesterol | LDL-4 | mg/dL |
| L5FC | Free cholesterol | LDL-5 | mg/dL |
| L6FC | Free cholesterol | LDL-6 | mg/dL |
| L1PL | Phospholipids | LDL-1 | mg/dL |
| L2PL | Phospholipids | LDL-2 | mg/dL |
| L3PL | Phospholipids | LDL-3 | mg/dL |
| L4PL | Phospholipids | LDL-4 | mg/dL |
| L5PL | Phospholipids | LDL-5 | mg/dL |
| L6PL | Phospholipids | LDL-6 | mg/dL |
| L1AB | Apo-B | LDL-1 | mg/dL |
| L2AB | Apo-B | LDL-2 | mg/dL |
| L3AB | Apo-B | LDL-3 | mg/dL |
| L4AB | Apo-B | LDL-4 | mg/dL |
| L5AB | Apo-B | LDL-5 | mg/dL |
| L6AB | Apo-B | LDL-5 | mg/dL |
| H1TG | Triglycerides | HDL-1 | mg/dL |
| H2TG | Triglycerides | HDL-2 | mg/dL |
| H3TG | Triglycerides | HDL-3 | mg/dL |
| H4TG | Triglycerides | HDL-4 | mg/dL |
| H1CH | Cholesterol | HDL-1 | mg/dL |
| H2CH | Cholesterol | HDL-2 | mg/dL |
| H3CH | Cholesterol | HDL-3 | mg/dL |
| H4CH | Cholesterol | HDL-4 | mg/dL |
| H1FC | Free cholesterol | HDL-1 | mg/dL |
| H2FC | Free cholesterol | HDL-2 | mg/dL |
| H3FC | Free cholesterol | HDL-3 | mg/dL |
| H4FC | Free cholesterol | HDL-4 | mg/dL |
| H1PL | Phospholipids | HDL-1 | mg/dL |
| H2PL | Phospholipids | HDL-2 | mg/dL |
| H3PL | Phospholipids | HDL-3 | mg/dL |
| H4PL | Phospholipids | HDL-4 | mg/dL |
| H1A1 | Apo-A1 | HDL-1 | mg/dL |
| H2A1 | Apo-A1 | HDL-2 | mg/dL |
| H3A1 | Apo-A1 | HDL-3 | mg/dL |
| H4A1 | Apo-A1 | HDL-4 | mg/dL |
| H1A2 | Apo-A2 | HDL-1 | mg/dL |
| H2A2 | Apo-A2 | HDL-2 | mg/dL |
| H3A2 | Apo-A2 | HDL-3 | mg/dL |
| H4A2 | Apo-A2 | HDL-4 | mg/dL |

LDL, low-density lipoprotein; VLDL, very-low-density lipoprotein; IDL, intermediate-density lipoprotein; HDL, high-density lipoprotein; LDL-C, LDL cholesterol; HDL-C, HDL cholesterol; Apo-A1, apolipoprotein A1; Apo-A2, apolipoprotein A2; Apo-B, apolipoprotein B.
