# Supplementary material for: Lipoprotein subfraction profiling in the search of new risk markers for myocardial infarction: The HUNT study
Source: PLoS One. 2023 May 5;18(5):e0285355. doi: 10.1371/journal.pone.0285355 (PMC10162525; doi:10.1371/journal.pone.0285355)
Supplement: S3 Table — (DOCX) [file pone.0285355.s008.docx]

S6 Table. Comparison of lipid variables between cases and controls (N=150)

| **Lipid variables and unit** | **Cases (n= 50)** | | **Controls (n= 100)** | | **p-value** |
| --- | --- | --- | --- | --- | --- |
|  | **Mean** | **Standard deviation** | **Mean** | **Standard deviation** |  |
| H4A1 (mg/dL) | 70.4 | 9.0 | 67.5 | 10.3 | 0.03 |
| H4A2 (mg/dL) | 16.8 | 2.6 | 15.9 | 3.4 | 0.05 |
| L5FC (mg/dL) | 6.4 | 1.6 | 5.9 | 1.5 | 0.05 |
| L4TG (mg/dL) | 3.3 | 0.9 | 3.0 | 1.0 | 0.06 |
| LDAB (mg/dL) | 87.6 | 17.8 | 83.1 | 13.1 | 0.08 |
| LDPN (nmol/L) | 1592.3 | 323.9 | 1511.2 | 237.5 | 0.08 |
| ABA1 (-/-) | 0.7 | 0.2 | 0.7 | 0.1 | 0.08 |
| H2CH (mg/dL) | 8.7 | 2.5 | 9.5 | 2.8 | 0.09 |
| H4PL (mg/dL) | 24.0 | 3.7 | 23.0 | 4.1 | 0.09 |
| L5CH (mg/dL) | 22.6 | 6.5 | 20.6 | 6.5 | 0.09 |
| L5PL (mg/dL) | 12.3 | 3.3 | 11.3 | 3.3 | 0.09 |
| H2FC (mg/dL) | 2.3 | 0.7 | 2.5 | 0.7 | 0.10 |
| H4CH (mg/dL) | 17.2 | 3.3 | 16.3 | 3.4 | 0.10 |
| L5AB (mg/dL) | 15.7 | 4.6 | 14.2 | 4.4 | 0.10 |
| H2PL (mg/dL) | 13.2 | 3.7 | 14.3 | 3.9 | 0.11 |
| L5PN (nmol/L) | 284.7 | 83.0 | 258.6 | 79.8 | 0.11 |
| L5PNmmol | 0.00028 | 0.00008 | 0.00026 | 0.00008 | 0.11 |
| LDTG (mg/dL) | 23.7 | 5.6 | 22.0 | 4.6 | 0.11 |
| TBPN (nmol/L) | 1926.4 | 348.2 | 1837.4 | 261.6 | 0.11 |
| TPAB (mg/dL) | 105.9 | 19.1 | 101.1 | 14.4 | 0.11 |
| L4AB (mg/dL) | 14.5 | 4.3 | 13.5 | 4.4 | 0.15 |
| L4PN (nmol/L) | 263.9 | 78.7 | 245.9 | 79.4 | 0.15 |
| LDHD (-/-) | 2.6 | 0.7 | 2.4 | 0.6 | 0.15 |
| L1TG (mg/dL) | 6.3 | 2.2 | 5.7 | 2.0 | 0.16 |
| H1A1 (mg/dL) | 25.8 | 14.1 | 29.0 | 15.7 | 0.17 |
| H1A2 (mg/dL) | 2.5 | 1.5 | 2.8 | 1.4 | 0.17 |
| L4PL (mg/dL) | 12.4 | 3.9 | 11.7 | 3.6 | 0.17 |
| L5TG (mg/dL) | 3.4 | 1.2 | 3.1 | 1.1 | 0.17 |
| LDPL (mg/dL) | 74.7 | 15.1 | 72.3 | 11.7 | 0.19 |
| H4FC (mg/dL) | 3.9 | 1.0 | 3.7 | 1.0 | 0.20 |
| H1PL (mg/dL) | 21.1 | 10.1 | 23.2 | 10.8 | 0.21 |
| H1TG (mg/dL) | 3.6 | 1.5 | 3.8 | 1.5 | 0.23 |
| H1FC (mg/dL) | 5.4 | 2.1 | 5.8 | 2.2 | 0.24 |
| L3TG (mg/dL) | 2.8 | 0.8 | 2.7 | 0.5 | 0.24 |
| L4CH (mg/dL) | 22.7 | 7.7 | 21.4 | 7.1 | 0.25 |
| H2TG (mg/dL) | 2.1 | 0.6 | 2.2 | 0.6 | 0.26 |
| H3CH (mg/dL) | 10.3 | 2.1 | 10.6 | 2.0 | 0.26 |
| HDPL (mg/dL) | 73.7 | 17.1 | 76.4 | 16.4 | 0.28 |
| L4FC (mg/dL) | 6.9 | 1.8 | 6.7 | 1.6 | 0.28 |
| HDFC (mg/dL) | 16.2 | 3.6 | 16.8 | 3.8 | 0.29 |
| LDFC (mg/dL) | 41.2 | 8.5 | 40.0 | 6.5 | 0.29 |
| H1CH (mg/dL) | 18.1 | 8.2 | 19.6 | 9.1 | 0.30 |
| H2A2 (mg/dL) | 3.5 | 1.0 | 3.7 | 1.1 | 0.30 |
| L6CH (mg/dL) | 25.6 | 9.2 | 23.5 | 7.3 | 0.30 |
| LDCH (mg/dL) | 135.6 | 30.3 | 131.3 | 23.9 | 0.30 |
| L6AB (mg/dL) | 21.1 | 8.0 | 19.3 | 6.6 | 0.31 |
| L6PN (nmol/L) | 384.4 | 145.9 | 351.2 | 119.8 | 0.31 |
| HDCH (mg/dL) | 54.4 | 13.2 | 56.3 | 13.7 | 0.35 |
| L6FC (mg/dL) | 6.6 | 2.1 | 6.2 | 1.7 | 0.35 |
| L6PL (mg/dL) | 14.2 | 4.6 | 13.2 | 3.6 | 0.37 |
| IDAB (mg/dL) | 6.3 | 2.3 | 5.9 | 2.1 | 0.38 |
| IDPN (nmol/L) | 115.1 | 42.4 | 107.7 | 37.8 | 0.38 |
| L6TG (mg/dL) | 4.5 | 1.7 | 4.2 | 1.3 | 0.40 |
| H2A1 (mg/dL) | 17.6 | 4.9 | 18.0 | 4.3 | 0.41 |
| H3A1 (mg/dL) | 27.4 | 5.0 | 28.1 | 4.9 | 0.45 |
| HDTG (mg/dL) | 11.2 | 2.9 | 11.5 | 3.1 | 0.45 |
| H3PL (mg/dL) | 15.6 | 3.3 | 16.0 | 3.2 | 0.51 |
| HDA1 (mg/dL) | 143.7 | 24.3 | 145.9 | 23.3 | 0.52 |
| H3A2 (mg/dL) | 6.6 | 1.2 | 6.8 | 1.3 | 0.53 |
| H3FC (mg/dL) | 2.5 | 0.7 | 2.5 | 0.6 | 0.53 |
| V5FC (mg/dL) | 0.6 | 0.6 | 0.6 | 0.4 | 0.56 |
| IDCH (mg/dL) | 16.4 | 6.6 | 15.6 | 6.1 | 0.61 |
| H3TG (mg/dL) | 2.3 | 0.7 | 2.4 | 0.7 | 0.63 |
| TPCH (mg/dL) | 235.4 | 34.6 | 231.9 | 27.0 | 0.66 |
| V4FC (mg/dL) | 2.8 | 1.3 | 2.8 | 1.3 | 0.66 |
| L2FC (mg/dL) | 6.5 | 2.4 | 6.8 | 2.5 | 0.69 |
| V1CH (mg/dL) | 9.1 | 6.8 | 9.4 | 6.7 | 0.69 |
| IDFC (mg/dL) | 4.5 | 2.0 | 4.3 | 1.8 | 0.71 |
| L2TG (mg/dL) | 2.9 | 0.8 | 2.7 | 0.6 | 0.71 |
| VLPL (mg/dL) | 24.0 | 9.6 | 24.3 | 10.7 | 0.71 |
| L1FC (mg/dL) | 7.8 | 2.1 | 7.7 | 2.1 | 0.72 |
| L2CH (mg/dL) | 18.9 | 8.5 | 19.7 | 8.9 | 0.72 |
| TPA1 (mg/dL) | 147.0 | 22.3 | 148.4 | 20.9 | 0.72 |
| VLCH (mg/dL) | 26.6 | 12.7 | 26.5 | 12.8 | 0.73 |
| VLFC (mg/dL) | 11.5 | 4.9 | 11.5 | 5.0 | 0.74 |
| V2TG (mg/dL) | 16.2 | 6.6 | 16.7 | 8.2 | 0.77 |
| L1AB (mg/dL) | 12.9 | 3.7 | 12.7 | 3.5 | 0.78 |
| L1PN (nmol/L) | 235.1 | 66.4 | 231.4 | 64.5 | 0.78 |
| HDA2 (mg/dL) | 30.6 | 4.1 | 30.6 | 4.1 | 0.80 |
| L2AB (mg/dL) | 10.6 | 4.3 | 10.9 | 4.4 | 0.80 |
| L2PL (mg/dL) | 10.6 | 4.3 | 11.0 | 4.4 | 0.80 |
| L2PN (nmol/L) | 193.6 | 77.6 | 198.9 | 79.3 | 0.80 |
| V2PL (mg/dL) | 3.9 | 1.7 | 4.0 | 2.1 | 0.81 |
| V3FC (mg/dL) | 2.1 | 1.2 | 2.1 | 1.2 | 0.83 |
| V4CH (mg/dL) | 6.0 | 2.6 | 5.7 | 2.7 | 0.85 |
| V3PL (mg/dL) | 4.7 | 2.0 | 4.7 | 2.2 | 0.86 |
| V2CH (mg/dL) | 4.0 | 2.2 | 4.0 | 2.2 | 0.87 |
| IDTG (mg/dL) | 13.6 | 9.9 | 14.0 | 11.1 | 0.88 |
| L3AB (mg/dL) | 12.9 | 4.3 | 12.8 | 3.7 | 0.88 |
| L3PN (nmol/L) | 235.1 | 78.3 | 232.1 | 66.7 | 0.88 |
| V2FC (mg/dL) | 1.9 | 1.1 | 1.9 | 1.2 | 0.89 |
| V3CH (mg/dL) | 4.7 | 2.4 | 4.6 | 2.5 | 0.90 |
| L3PL (mg/dL) | 12.0 | 4.1 | 11.9 | 3.5 | 0.91 |
| L1PL (mg/dL) | 13.6 | 3.8 | 13.6 | 3.7 | 0.92 |
| TPA2 (mg/dL) | 30.3 | 4.3 | 30.3 | 4.2 | 0.92 |
| V5PL (mg/dL) | 2.1 | 0.7 | 2.1 | 0.7 | 0.92 |
| V5TG (mg/dL) | 3.4 | 0.8 | 3.5 | 0.7 | 0.92 |
| V3TG (mg/dL) | 14.8 | 5.7 | 14.9 | 6.7 | 0.93 |
| V1TG (mg/dL) | 46.3 | 32.4 | 48.2 | 37.4 | 0.94 |
| VLTG (mg/dL) | 93.7 | 47.2 | 95.3 | 53.8 | 0.94 |
| H4TG (mg/dL) | 3.4 | 0.9 | 3.3 | 1.1 | 0.96 |
| TPTG (mg/dL) | 149.4 | 67.5 | 150.1 | 71.9 | 0.96 |
| V4TG (mg/dL) | 11.0 | 3.8 | 10.8 | 3.8 | 0.96 |
| V1PL (mg/dL) | 7.2 | 4.7 | 7.5 | 5.6 | 0.97 |
| V4PL (mg/dL) | 5.6 | 1.9 | 5.4 | 2.0 | 0.97 |
| VLAB (mg/dL) | 10.3 | 3.9 | 10.1 | 4.1 | 0.97 |
| VLPN (nmol/L) | 187.0 | 70.2 | 183.7 | 73.7 | 0.97 |
| L3CH (mg/dL) | 21.7 | 8.2 | 21.6 | 7.1 | 0.98 |
| L3FC (mg/dL) | 7.1 | 2.0 | 7.1 | 1.9 | 0.98 |
| V1FC (mg/dL) | 2.9 | 2.3 | 3.0 | 2.6 | 0.98 |
| V5CH (mg/dL) | 1.4 | 0.6 | 1.4 | 0.6 | 0.98 |
| L1CH (mg/dL) | 24.0 | 7.3 | 24.0 | 7.1 | 0.99 |
| IDPL (mg/dL) | 8.7 | 3.3 | 8.7 | 3.5 | 1.00 |

L or LD, low-density lipoprotein; V, very-low-density lipoprotein; I, intermediate-density lipoprotein; H, high-density lipoprotein; CH, cholesterol; TG, triglycerides; FC, free cholesterol; PL, phospholipid; PN, particle number; A1, apolipoprotein A1; A2, apolipoprotein A2; AB, apolipoprotein B; TP, total plasma.
