# Supplementary material for: Lipoprotein subfraction profiling in the search of new risk markers for myocardial infarction: The HUNT study
Source: PLoS One. 2023 May 5;18(5):e0285355. doi: 10.1371/journal.pone.0285355 (PMC10162525; doi:10.1371/journal.pone.0285355)
Supplement: S4 Table — (DOCX) [file pone.0285355.s009.docx]

S8 Table. Comparison of lipid variables levels between male cases and male controls (N= 90)

| **Lipid variables and unit** | **Cases (n= 30)** | | **Controls (n= 60)** | | **p-value** |
| --- | --- | --- | --- | --- | --- |
|  | **Mean** | **Standard deviation** | **Mean** | **Standard deviation** |  |
| ABA1 (-/-) | 0.78 | 0.14 | 0.72 | 0.15 | 0.084 |
| H1A1 (mg/dL) | 19.40 | 7.89 | 25.23 | 13.64 | 0.066 |
| H1A2 (mg/dL) | 1.89 | 0.87 | 2.49 | 1.23 | 0.019 |
| H1CH (mg/dL) | 14.37 | 4.70 | 17.15 | 7.49 | 0.132 |
| H1FC (mg/dL) | 4.54 | 1.26 | 5.22 | 1.94 | 0.163 |
| H1PL (mg/dL) | 16.37 | 5.63 | 20.28 | 9.07 | 0.062 |
| H1TG (mg/dL) | 2.97 | 0.99 | 3.62 | 1.50 | 0.046 |
| H2A1 (mg/dL) | 15.44 | 3.25 | 17.01 | 3.75 | 0.056 |
| H2A2 (mg/dL) | 3.10 | 0.90 | 3.55 | 1.00 | 0.026 |
| H2CH (mg/dL) | 7.53 | 2.00 | 8.62 | 2.58 | 0.055 |
| H2FC (mg/dL) | 2.01 | 0.54 | 2.23 | 0.64 | 0.081 |
| H2PL (mg/dL) | 11.49 | 2.93 | 13.21 | 3.56 | 0.037 |
| H2TG (mg/dL) | 1.92 | 0.48 | 2.17 | 0.62 | 0.075 |
| H3A1 (mg/dL) | 25.43 | 4.13 | 26.95 | 4.62 | 0.199 |
| H3A2 (mg/dL) | 6.26 | 1.18 | 6.67 | 1.27 | 0.160 |
| H3CH (mg/dL) | 9.38 | 1.78 | 9.96 | 1.81 | 0.121 |
| H3FC (mg/dL) | 2.23 | 0.51 | 2.30 | 0.53 | 0.366 |
| H3PL (mg/dL) | 14.14 | 2.81 | 15.04 | 2.74 | 0.172 |
| H3TG (mg/dL) | 2.22 | 0.57 | 2.39 | 0.66 | 0.219 |
| H4A1 (mg/dL) | 69.55 | 8.20 | 66.61 | 9.40 | 0.043 |
| H4A2 (mg/dL) | 16.82 | 2.53 | 15.86 | 3.11 | 0.068 |
| H4CH (mg/dL) | 16.80 | 2.98 | 15.75 | 3.15 | 0.083 |
| H4FC (mg/dL) | 3.72 | 0.86 | 3.48 | 0.99 | 0.202 |
| H4PL (mg/dL) | 23.34 | 3.51 | 22.38 | 4.05 | 0.158 |
| H4TG (mg/dL) | 3.49 | 0.77 | 3.49 | 1.04 | 0.546 |
| HDA1 (mg/dL) | 132.13 | 15.97 | 138.74 | 20.69 | 0.148 |
| HDA2 (mg/dL) | 29.25 | 3.66 | 29.88 | 3.90 | 0.518 |
| HDCH (mg/dL) | 48.37 | 9.08 | 51.83 | 11.62 | 0.166 |
| HDFC (mg/dL) | 14.63 | 2.49 | 15.71 | 3.61 | 0.185 |
| HDPL (mg/dL) | 65.26 | 11.56 | 70.74 | 13.82 | 0.074 |
| HDTG (mg/dL) | 10.42 | 2.31 | 11.48 | 2.98 | 0.103 |
| IDAB (mg/dL) | 6.20 | 2.07 | 6.11 | 2.00 | 0.854 |
| IDCH (mg/dL) | 16.13 | 6.00 | 15.96 | 5.73 | 0.956 |
| IDFC (mg/dL) | 4.43 | 1.72 | 4.43 | 1.71 | 0.922 |
| IDPL (mg/dL) | 8.56 | 2.79 | 9.09 | 3.58 | 0.638 |
| IDPN (nmol/L) | 112.80 | 37.69 | 111.01 | 36.36 | 0.851 |
| IDTG (mg/dL) | 13.90 | 8.69 | 16.18 | 11.80 | 0.614 |
| L1AB (mg/dL) | 11.67 | 3.31 | 11.33 | 3.22 | 0.572 |
| L1CH (mg/dL) | 21.70 | 6.68 | 21.26 | 6.33 | 0.681 |
| L1FC (mg/dL) | 7.06 | 1.96 | 6.88 | 1.75 | 0.543 |
| L1PL (mg/dL) | 12.34 | 3.41 | 12.10 | 3.33 | 0.669 |
| L1PN (nmol/L) | 212.22 | 60.19 | 205.94 | 58.45 | 0.572 |
| L1TG (mg/dL) | 5.74 | 1.87 | 5.46 | 1.97 | 0.421 |
| L2AB (mg/dL) | 9.72 | 3.85 | 9.66 | 3.62 | 0.905 |
| L2CH (mg/dL) | 17.04 | 7.64 | 17.16 | 7.35 | 0.986 |
| L2FC (mg/dL) | 5.83 | 2.01 | 5.90 | 2.04 | 0.945 |
| L2PL (mg/dL) | 9.62 | 3.86 | 9.65 | 3.64 | 0.949 |
| L2PN (nmol/L) | 176.77 | 70.09 | 175.57 | 65.89 | 0.905 |
| L2TG (mg/dL) | 2.66 | 0.70 | 2.58 | 0.63 | 0.510 |
| L3AB (mg/dL) | 12.46 | 4.34 | 12.03 | 3.54 | 0.687 |
| L3CH (mg/dL) | 20.83 | 8.36 | 20.20 | 6.80 | 0.732 |
| L3FC (mg/dL) | 6.67 | 1.89 | 6.55 | 1.71 | 0.837 |
| L3PL (mg/dL) | 11.53 | 4.12 | 11.15 | 3.33 | 0.638 |
| L3PN (nmol/L) | 226.49 | 78.98 | 218.70 | 64.28 | 0.687 |
| L3TG (mg/dL) | 2.62 | 0.62 | 2.53 | 0.54 | 0.308 |
| L4AB (mg/dL) | 15.12 | 4.52 | 13.99 | 4.40 | 0.193 |
| L4CH (mg/dL) | 23.69 | 7.88 | 21.95 | 7.33 | 0.215 |
| L4FC (mg/dL) | 7.00 | 1.77 | 6.62 | 1.71 | 0.243 |
| L4PL (mg/dL) | 12.85 | 4.02 | 11.96 | 3.73 | 0.190 |
| L4PN (nmol/L) | 274.85 | 82.26 | 254.44 | 80.02 | 0.190 |
| L4TG (mg/dL) | 3.38 | 0.96 | 3.05 | 1.03 | 0.204 |
| L5AB (mg/dL) | 16.61 | 4.40 | 15.17 | 4.29 | 0.289 |
| L5CH (mg/dL) | 24.08 | 6.28 | 22.08 | 6.37 | 0.208 |
| L5FC (mg/dL) | 6.57 | 1.52 | 6.06 | 1.50 | 0.138 |
| L5PL (mg/dL) | 13.00 | 3.15 | 12.01 | 3.24 | 0.223 |
| L5PN (nmol/L) | 301.95 | 80.01 | 275.85 | 77.92 | 0.296 |
| L5PNmmol |  |  |  |  | 0.296 |
| L5TG (mg/dL) | 3.60 | 1.21 | 3.28 | 1.09 | 0.436 |
| L6AB (mg/dL) | 21.54 | 7.03 | 20.24 | 6.91 | 0.431 |
| L6CH (mg/dL) | 26.10 | 8.05 | 24.40 | 7.59 | 0.507 |
| L6FC (mg/dL) | 6.57 | 1.74 | 6.22 | 1.72 | 0.496 |
| L6PL (mg/dL) | 14.23 | 3.92 | 13.59 | 3.73 | 0.641 |
| L6PN (nmol/L) | 391.56 | 127.92 | 367.96 | 125.56 | 0.429 |
| L6TG (mg/dL) | 4.56 | 1.42 | 4.27 | 1.24 | 0.449 |
| LDAB (mg/dL) | 86.65 | 16.93 | 81.71 | 13.43 | 0.130 |
| LDCH (mg/dL) | 133.38 | 29.35 | 127.29 | 23.45 | 0.215 |
| LDFC (mg/dL) | 40.08 | 7.88 | 38.60 | 6.33 | 0.221 |
| LDHD (-/-) | 2.81 | 0.66 | 2.56 | 0.66 | 0.091 |
| LDPL (mg/dL) | 73.22 | 14.78 | 69.99 | 11.35 | 0.168 |
| LDPN (nmol/L) | 1575.49 | 307.82 | 1485.79 | 244.27 | 0.130 |
| LDTG (mg/dL) | 23.07 | 5.11 | 21.86 | 4.58 | 0.483 |
| TBPN (nmol/L) | 1912.66 | 325.97 | 1824.81 | 274.70 | 0.234 |
| TPA1 (mg/dL) | 136.47 | 14.89 | 142.59 | 19.13 | 0.190 |
| TPA2 (mg/dL) | 28.79 | 3.80 | 29.50 | 4.00 | 0.521 |
| TPAB (mg/dL) | 105.19 | 17.93 | 100.36 | 15.11 | 0.238 |
| TPCH (mg/dL) | 227.34 | 32.99 | 225.42 | 25.92 | 0.778 |
| TPTG (mg/dL) | 151.74 | 56.85 | 164.84 | 74.08 | 0.538 |
| V1CH (mg/dL) | 9.76 | 5.94 | 10.89 | 6.80 | 0.426 |
| V1FC (mg/dL) | 3.11 | 1.90 | 3.59 | 2.71 | 0.662 |
| V1PL (mg/dL) | 7.83 | 3.91 | 8.91 | 5.79 | 0.638 |
| V1TG (mg/dL) | 49.43 | 26.36 | 57.66 | 39.80 | 0.590 |
| V2CH (mg/dL) | 4.22 | 2.16 | 4.27 | 2.04 | 0.678 |
| V2FC (mg/dL) | 2.05 | 1.04 | 2.13 | 1.08 | 0.739 |
| V2PL (mg/dL) | 4.18 | 1.60 | 4.33 | 1.97 | 0.669 |
| V2TG (mg/dL) | 17.05 | 6.31 | 18.02 | 7.97 | 0.535 |
| V3CH (mg/dL) | 4.91 | 2.29 | 4.92 | 2.28 | 0.761 |
| V3FC (mg/dL) | 2.21 | 1.05 | 2.33 | 1.15 | 0.578 |
| V3PL (mg/dL) | 4.94 | 1.75 | 5.12 | 2.09 | 0.611 |
| V3TG (mg/dL) | 15.33 | 5.28 | 15.89 | 6.36 | 0.584 |
| V4CH (mg/dL) | 5.86 | 2.61 | 5.72 | 2.51 | 0.857 |
| V4FC (mg/dL) | 2.77 | 1.17 | 2.86 | 1.15 | 0.464 |
| V4PL (mg/dL) | 5.52 | 1.60 | 5.60 | 1.79 | 0.669 |
| V4TG (mg/dL) | 10.80 | 2.80 | 11.19 | 3.58 | 0.502 |
| V5CH (mg/dL) | 1.24 | 0.54 | 1.32 | 0.68 | 0.817 |
| V5FC (mg/dL) | 0.57 | 0.45 | 0.67 | 0.43 | 0.210 |
| V5PL (mg/dL) | 1.99 | 0.55 | 2.12 | 0.69 | 0.635 |
| V5TG (mg/dL) | 3.25 | 0.60 | 3.48 | 0.74 | 0.406 |
| VLAB (mg/dL) | 10.53 | 3.16 | 10.91 | 3.75 | 0.558 |
| VLCH (mg/dL) | 27.49 | 11.62 | 28.63 | 11.55 | 0.529 |
| VLFC (mg/dL) | 11.78 | 4.26 | 12.47 | 4.71 | 0.451 |
| VLPL (mg/dL) | 24.97 | 8.17 | 26.79 | 10.26 | 0.346 |
| VLPN (nmol/L) | 191.38 | 57.39 | 198.43 | 68.09 | 0.555 |
| VLTG (mg/dL) | 97.80 | 39.11 | 107.77 | 54.86 | 0.543 |

L or LD, low-density lipoprotein; V, very-low-density lipoprotein; I, intermediate-density lipoprotein; H, high-density lipoprotein; CH, cholesterol; TG, triglycerides; FC, free cholesterol; PL, phospholipid; PN, particle number; A1, apolipoprotein A1; A2, apolipoprotein A2; AB, apolipoprotein B; TP, total plasma.
