# Supplementary material for: Lipoprotein subfraction profiling in the search of new risk markers for myocardial infarction: The HUNT study
Source: PLoS One. 2023 May 5;18(5):e0285355. doi: 10.1371/journal.pone.0285355 (PMC10162525; doi:10.1371/journal.pone.0285355)
Supplement: S5 Table — (DOCX) [file pone.0285355.s010.docx]

S9 Table. Comparison of lipid variables between cases and controls in a 2-year observation period (N= 57)

| **Lipid variables and unit** | **Cases (n= 19)** | | **Controls (n= 38)** | | **p-value** |
| --- | --- | --- | --- | --- | --- |
|  | **Mean** | **Standard deviation** | **Mean** | **Standard deviation** |  |
| ABA1 (-/-) | 0.74 | 0.19 | 0.68 | 0.16 | 0.267 |
| H1A1 (mg/dL) | 29.54 | 19.33 | 32.54 | 16.06 | 0.229 |
| H1A2 (mg/dL) | 2.99 | 2.03 | 3.06 | 1.38 | 0.431 |
| H1CH (mg/dL) | 19.98 | 10.89 | 21.47 | 8.66 | 0.236 |
| H1FC (mg/dL) | 5.89 | 2.87 | 6.26 | 2.17 | 0.264 |
| H1PL (mg/dL) | 23.93 | 13.63 | 25.42 | 10.77 | 0.335 |
| H1TG (mg/dL) | 4.14 | 1.40 | 4.00 | 1.61 | 0.735 |
| H2A1 (mg/dL) | 18.87 | 6.19 | 18.61 | 4.14 | 0.892 |
| H2A2 (mg/dL) | 3.67 | 1.05 | 3.78 | 0.93 | 0.642 |
| H2CH (mg/dL) | 8.99 | 3.25 | 9.94 | 2.81 | 0.220 |
| H2FC (mg/dL) | 2.42 | 0.88 | 2.56 | 0.68 | 0.330 |
| H2PL (mg/dL) | 14.03 | 4.70 | 14.87 | 3.97 | 0.531 |
| H2TG (mg/dL) | 2.23 | 0.48 | 2.21 | 0.68 | 0.710 |
| H3A1 (mg/dL) | 27.78 | 5.88 | 28.20 | 4.93 | 0.600 |
| H3A2 (mg/dL) | 6.62 | 1.38 | 6.82 | 1.21 | 0.654 |
| H3CH (mg/dL) | 10.32 | 2.67 | 10.76 | 1.94 | 0.343 |
| H3FC (mg/dL) | 2.53 | 0.80 | 2.56 | 0.55 | 0.773 |
| H3PL (mg/dL) | 16.02 | 4.02 | 16.01 | 2.85 | 0.939 |
| H3TG (mg/dL) | 2.40 | 0.60 | 2.30 | 0.72 | 0.451 |
| H4A1 (mg/dL) | 68.61 | 8.52 | 66.80 | 10.26 | 0.407 |
| H4A2 (mg/dL) | 15.86 | 2.53 | 15.49 | 3.65 | 0.515 |
| H4CH (mg/dL) | 16.31 | 2.79 | 16.10 | 3.51 | 0.710 |
| H4FC (mg/dL) | 3.80 | 0.93 | 3.64 | 1.06 | 0.565 |
| H4PL (mg/dL) | 23.68 | 3.61 | 22.55 | 3.88 | 0.302 |
| H4TG (mg/dL) | 3.41 | 1.06 | 3.20 | 1.16 | 0.446 |
| HDA1 (mg/dL) | 146.41 | 31.28 | 149.69 | 22.89 | 0.467 |
| HDA2 (mg/dL) | 30.25 | 4.75 | 30.63 | 3.75 | 0.666 |
| HDCH (mg/dL) | 55.76 | 16.73 | 58.80 | 13.35 | 0.260 |
| HDFC (mg/dL) | 17.08 | 4.76 | 17.61 | 4.23 | 0.402 |
| HDPL (mg/dL) | 77.72 | 21.67 | 78.80 | 16.34 | 0.710 |
| HDTG (mg/dL) | 12.09 | 2.17 | 11.53 | 3.31 | 0.361 |
| IDAB (mg/dL) | 6.65 | 2.16 | 5.92 | 2.18 | 0.184 |
| IDCH (mg/dL) | 16.58 | 6.00 | 15.69 | 6.38 | 0.672 |
| IDFC (mg/dL) | 4.51 | 1.74 | 4.29 | 1.90 | 0.617 |
| IDPL (mg/dL) | 8.49 | 2.73 | 8.78 | 3.63 | 0.892 |
| IDPN (nmol/L) | 120.90 | 39.38 | 107.71 | 39.66 | 0.187 |
| IDTG (mg/dL) | 12.88 | 6.67 | 13.87 | 11.89 | 0.761 |
| L1AB (mg/dL) | 13.49 | 3.32 | 12.98 | 3.45 | 0.648 |
| L1CH (mg/dL) | 24.37 | 6.16 | 24.83 | 6.89 | 0.748 |
| L1FC (mg/dL) | 8.01 | 1.84 | 8.01 | 1.98 | 0.933 |
| L1PL (mg/dL) | 14.02 | 3.29 | 13.94 | 3.53 | 0.906 |
| L1PN (nmol/L) | 245.23 | 60.43 | 235.96 | 62.72 | 0.648 |
| L1TG (mg/dL) | 6.84 | 1.74 | 5.60 | 1.90 | 0.016 |
| L2AB (mg/dL) | 9.67 | 3.25 | 11.32 | 5.04 | 0.264 |
| L2CH (mg/dL) | 16.58 | 6.49 | 20.68 | 10.18 | 0.170 |
| L2FC (mg/dL) | 5.92 | 1.94 | 7.03 | 2.86 | 0.275 |
| L2PL (mg/dL) | 9.63 | 3.31 | 11.48 | 5.04 | 0.264 |
| L2PN (nmol/L) | 175.91 | 59.18 | 205.77 | 91.60 | 0.264 |
| L2TG (mg/dL) | 3.03 | 0.70 | 2.77 | 0.63 | 0.322 |
| L3AB (mg/dL) | 12.15 | 3.75 | 13.13 | 4.04 | 0.318 |
| L3CH (mg/dL) | 19.83 | 7.03 | 22.43 | 7.97 | 0.217 |
| L3FC (mg/dL) | 6.68 | 1.68 | 7.33 | 2.12 | 0.187 |
| L3PL (mg/dL) | 11.24 | 3.51 | 12.28 | 3.93 | 0.352 |
| L3PN (nmol/L) | 220.96 | 68.21 | 238.80 | 73.47 | 0.318 |
| L3TG (mg/dL) | 3.11 | 0.80 | 2.73 | 0.52 | 0.067 |
| L4AB (mg/dL) | 14.29 | 4.60 | 13.64 | 4.50 | 0.697 |
| L4CH (mg/dL) | 21.87 | 7.77 | 21.72 | 7.51 | 0.852 |
| L4FC (mg/dL) | 6.78 | 1.70 | 6.79 | 1.75 | 0.859 |
| L4PL (mg/dL) | 12.13 | 3.96 | 11.84 | 3.82 | 0.946 |
| L4PN (nmol/L) | 259.79 | 83.55 | 248.05 | 81.78 | 0.697 |
| L4TG (mg/dL) | 3.68 | 1.05 | 2.96 | 1.11 | 0.025 |
| L5AB (mg/dL) | 16.50 | 5.55 | 14.15 | 4.85 | 0.190 |
| L5CH (mg/dL) | 23.38 | 7.48 | 20.62 | 7.07 | 0.176 |
| L5FC (mg/dL) | 6.58 | 1.74 | 5.89 | 1.65 | 0.150 |
| L5PL (mg/dL) | 12.76 | 3.74 | 11.26 | 3.57 | 0.143 |
| L5PN (nmol/L) | 300.02 | 100.81 | 257.21 | 88.10 | 0.192 |
| L5TG (mg/dL) | 3.95 | 1.53 | 3.02 | 1.25 | 0.049 |
| L6AB (mg/dL) | 23.16 | 9.72 | 18.83 | 6.94 | 0.139 |
| L6CH (mg/dL) | 27.91 | 11.77 | 23.16 | 7.85 | 0.236 |
| L6FC (mg/dL) | 7.13 | 2.72 | 6.14 | 1.83 | 0.236 |
| L6PL (mg/dL) | 15.43 | 5.89 | 13.08 | 3.78 | 0.233 |
| L6PN (nmol/L) | 421.06 | 176.78 | 342.39 | 126.16 | 0.141 |
| L6TG (mg/dL) | 5.28 | 2.22 | 4.06 | 1.31 | 0.021 |
| LDAB (mg/dL) | 89.53 | 18.76 | 83.62 | 13.88 | 0.250 |
| LDCH (mg/dL) | 133.90 | 25.02 | 133.94 | 25.78 | 0.852 |
| LDFC (mg/dL) | 41.13 | 7.31 | 41.01 | 7.14 | 0.866 |
| LDHD (-/-) | 2.58 | 0.82 | 2.39 | 0.67 | 0.431 |
| LDPL (mg/dL) | 74.92 | 13.12 | 73.40 | 12.34 | 0.774 |
| LDPN (nmol/L) | 1627.96 | 341.14 | 1520.48 | 252.43 | 0.250 |
| LDTG (mg/dL) | 25.81 | 6.31 | 21.67 | 4.59 | 0.021 |
| TBPN (nmol/L) | 1960.86 | 365.30 | 1835.94 | 258.56 | 0.170 |
| TPA1 (mg/dL) | 149.97 | 27.87 | 152.05 | 20.53 | 0.520 |
| TPA2 (mg/dL) | 29.81 | 4.94 | 30.39 | 3.89 | 0.588 |
| TPAB (mg/dL) | 107.84 | 20.09 | 100.97 | 14.22 | 0.170 |
| TPCH (mg/dL) | 237.07 | 32.44 | 236.41 | 22.34 | 0.748 |
| TPTG (mg/dL) | 145.78 | 48.29 | 148.21 | 74.91 | 0.710 |
| V1CH (mg/dL) | 8.51 | 5.36 | 9.14 | 6.92 | 0.892 |
| V1FC (mg/dL) | 2.60 | 1.84 | 2.92 | 2.73 | 1.000 |
| V1PL (mg/dL) | 6.52 | 3.72 | 7.36 | 5.94 | 0.892 |
| V1TG (mg/dL) | 41.11 | 23.06 | 47.40 | 39.45 | 0.866 |
| V2CH (mg/dL) | 4.15 | 2.23 | 3.85 | 2.23 | 0.691 |
| V2FC (mg/dL) | 1.83 | 1.10 | 1.83 | 1.16 | 0.986 |
| V2PL (mg/dL) | 3.89 | 1.72 | 3.80 | 2.19 | 0.774 |
| V2TG (mg/dL) | 16.23 | 6.18 | 16.24 | 8.76 | 0.774 |
| V3CH (mg/dL) | 4.64 | 2.53 | 4.39 | 2.54 | 0.729 |
| V3FC (mg/dL) | 2.01 | 1.14 | 2.03 | 1.25 | 0.986 |
| V3PL (mg/dL) | 4.58 | 2.06 | 4.55 | 2.36 | 1.000 |
| V3TG (mg/dL) | 14.49 | 6.02 | 14.40 | 7.07 | 0.879 |
| V4CH (mg/dL) | 6.33 | 2.71 | 5.64 | 2.77 | 0.498 |
| V4FC (mg/dL) | 2.86 | 1.15 | 2.78 | 1.38 | 0.912 |
| V4PL (mg/dL) | 5.67 | 1.80 | 5.39 | 2.14 | 0.748 |
| V4TG (mg/dL) | 10.79 | 3.48 | 10.64 | 4.08 | 0.787 |
| V5CH (mg/dL) | 1.40 | 0.66 | 1.39 | 0.65 | 0.446 |
| V5FC (mg/dL) | 0.62 | 0.44 | 0.63 | 0.43 | 0.966 |
| V5PL (mg/dL) | 2.09 | 0.75 | 2.11 | 0.73 | 0.426 |
| V5TG (mg/dL) | 3.52 | 0.70 | 3.56 | 0.88 | 0.588 |
| VLAB (mg/dL) | 10.11 | 3.53 | 9.87 | 4.20 | 0.806 |
| VLCH (mg/dL) | 26.32 | 11.59 | 26.05 | 13.15 | 0.986 |
| VLFC (mg/dL) | 11.21 | 4.33 | 11.35 | 5.16 | 0.959 |
| VLPL (mg/dL) | 23.22 | 9.06 | 23.95 | 11.38 | 0.839 |
| VLPN (nmol/L) | 183.75 | 64.20 | 179.52 | 76.34 | 0.813 |
| VLTG (mg/dL) | 88.18 | 36.92 | 93.35 | 57.02 | 0.973 |

L or LD, low-density lipoprotein; V, very-low-density lipoprotein; I, intermediate-density lipoprotein; H, high-density lipoprotein; CH, cholesterol; TG, triglycerides; FC, free cholesterol; PL, phospholipid; PN, particle number; A1, apolipoprotein A1; A2, apolipoprotein A2; AB, apolipoprotein B; TP, total plasma.
